# Fluid-phase and membrane markers reveal spatio-temporal dynamics of membrane traffic and repair in the green alga *Chara australis*

**DOI:** 10.1007/s00709-021-01627-z

**Published:** 2021-03-11

**Authors:** Aniela Sommer, Margit Hoeftberger, Ilse Foissner

**Affiliations:** Department of Biosciences, University of Salzburg, Hellbrunnerstr. 34, 5020 Salzburg, Austria

**Keywords:** Alexa 488 hydrazide, *Chara* internodal cell, FM4-64, Live-cell imaging, Endocytosis markers, Fluid-phase endocytosis, Plant endocytosis

## Abstract

We investigated the mechanisms and the spatio-temporal dynamics of fluid-phase and membrane internalization in the green alga *Chara australis* using fluorescent hydrazides markers alone, or in conjunction with styryl dyes**.** Using live-cell imaging, immunofluorescence and inhibitor studies we revealed that both fluid-phase and membrane dyes were actively taken up into the cytoplasm by clathrin-mediated endocytosis and stained various classes of endosomes including brefeldin A- and wortmannin-sensitive organelles (trans-Golgi network and multivesicular bodies). Uptake of fluorescent hydrazides was poorly sensitive to cytochalasin D, suggesting that actin plays a minor role in constitutive endocytosis in *Chara* internodal cells. Sequential pulse-labelling experiments revealed novel aspects of the temporal progression of endosomes in *Chara* internodal cells. The internalized fluid-phase marker distributed to early compartments within 10 min from dye exposure and after about 30 min, it was found almost exclusively in late endocytic compartments. Notably, fluid cargo consecutively internalized at time intervals of more than 1h, was not targeted to the same vesicular structures, but was sorted into distinct late compartments. We further found that fluorescent hydrazide dyes distributed not only to rapidly recycling endosomes but also to long-lived compartments that participated in plasma membrane repair after local laser injury. Our approach highlights the benefits of combining different fluid-phase markers in conjunction with membrane dyes in simultaneous and sequential application modus for investigating vesicle traffic, especially in organisms, which are still refractory to genetic transformation like characean algae.

**Supplementary Information:**

The online version contains supplementary material available at 10.1007/s00709-021-01627-z.

## Introduction

Eukaryotic cells internalize plasma membrane and extracellular material by endocytosis. During this process, the plasma membrane produces an invagination, which pinches off to form an endocytic vesicle. Endocytic vesicles then fuse with each other and with other organelles for degradation of membranes and vesicle contents, and for recycling. Endocytosis is not only important for the recycling of plasma membrane components and for nutrient uptake, but also for signaling in animal and plant cells (see Geldner et al. 2007; Doherty and McMahon 2009; Irani and Russinova 2009; Baisa et al. 2013; and references therein). Our knowledge about endocytosis in general is largely derived from animal and fungal cells, in which a variety of endocytic pathways have been described. In recent years, much progress was made also in plant cell research, with the introduction of fluorescent dyes as endocytic tracers, and with the development of new molecular biological techniques (for recent reviews see Reynolds et al. 2018; Rosquete et al. 2018; Rodriguez-Furlan et al. 2019).

Endocytosis can be classified according to the cargo (e. g. receptor-mediated endocytosis and fluid phase endocytosis - FPE, including uptake of assimilates or nutrients), or according to the mechanism of endocytosis, i. e. the machinery which is used to form an endocytic vesicle from the plasma membrane (Doherty and McMahon 2009; Lundmark and Carlsson 2010). Receptor-mediated endocytosis and fluid-phase endocytosis do not exclude each other, and receptor-dependent endocytosis has often been found to be accompanied by uptake of extracellular cargo (Bandmann et al. 2014). Furthermore, most cargos can enter by several pathways, and it has therefore been suggested to distinguish endocytosis according to their differential dependence on certain lipids and proteins (Doherty and McMahon 2009). Among these, clathrin-mediated endocytosis is best defined and involves membrane curvature by clathrin triskelions, followed by dynamin-mediated membrane fission (Lundmark and Carlsson 2010). Coated pits and coated vesicles at or near the plasma membrane of plant cells, including characean internodes (Pickett-Heaps 1967) have been observed on electron microscopical sections already in the 1960s (see Newcomb 1980 for further references), and later research confirmed the existence of clathrin-dependent endocytosis in plant cells (e.g. Baisa et al. 2013 and references therein). Contradictory data exist about the requirement for actin or microtubules in clathrin-mediated endocytosis (Baisa et al. 2013; Narasimhan et al. 2020). Alternative, clathrin-independent endocytic pathways have also been reported in plant cells (e.g. Grebe et al. 2003; Moscatelli et al. 2007; Onelli et al. 2008; Boutte and Grebe 2009; Bandmann and Homann 2012; Baral et al. 2015, review by Fan et al. 2015). Most of these pathways are likely to be sterol-dependent, but more research is required for elucidating the mechanism by which endocytic vesicles are formed and recognize their target organelles.

The process of endocytosis can be followed *in vivo* with the fluorescence or confocal laser scanning microscope, using plasma membrane markers or fluid-phase dyes which are taken up together with the extracellular medium. Among the membrane markers, FM styryl dyes are the most frequently used, although possible side effects should be considered (Meckel et al. 2004; Jelinkova et al. 2010). These dyes incorporate into the plasma membrane via a hydrophobic hydrocarbon tail, whereas their hydrophilic head group prevents passage across membranes. Time-course studies showed early co-localization of FM-dye signal with TGN specific markers, suggesting that after uncoating, the endocytic vesicles fuse firstly with the TGN, the early endosome in plant cells (Dettmer et al. 2006; Foissner and Wasteneys 2007). From there, the FM-styryl dye is further distributed to MVBs, and finally becomes part of the lytic vacuole membrane. In-depth studies using endocytic organelle- or cargo-specific markers are however only possible in and restricted to organisms that can easily be genetically transformed.

The endocytic uptake of external medium in intact plant cells can be followed using fluorescent markers that are plasma membrane impermeant, but are able to pass through the cell wall. Fluorescent hydrazides were introduced as fluid-phase markers for plant cell endocytosis by Oparka and coworkers (Oparka 1991). Meanwhile, fluorescent dextrans, Coro-Na, fluorescein isothiocyanate labelled-bovine serum albumin and Alexa hydrazides were also successfully applied to study the uptake of extracellular fluid by heterotrophic, walled plant cells (Emans et al. 2002; Etxeberria et al. 2006, 2007a, b, 2012; Gall et al. 2010; Adlassnig et al. 2012; Bandmann et al. 2014).

A wide variety of heterotrophic plant cells and tissues was reported to use FPE as a distinct, clathrin-independent mechanism for the uptake of nutrients, extracellular matrix components and sugar analogs (Etxeberria et al. 2012), yet a detailed characterization of this endocytic pathway is still missing. Comparatively little is known about the role of FPE in autotrophic cells, where uptake of nutrients from the cell exterior is less important, but FPE might be required for other cellular processes like cell wall homeostasis, plasma membrane recycling and heavy metal uptake and sequestration (Baluska et al. 2005; Illes et al. 2006; Wang et al. 2014).

In the current study, we investigated the mechanisms (clathrin-dependent or not) and the spatio-temporal dynamics of fluid-phase and membrane internalization in internodal cells of the branched, multi-cellular green alga *Chara australis*, with focus on FPE. We conducted pulse-chase experiments with fluorescent hydrazides as extracellular medium markers alone or in conjunction with FM styryl dyes, applied simultaneously or consecutively, and followed their spatio-temporal distribution to target organelles. Immunofluorescence and pharmacological tools as well as metabolic inhibitors were used in order to identify the endosomal compartments and, respectively, to unravel the uptake and internalization mechanisms involved in the endocytic pathway of *Chara*.

## Materials and methods

### Algal material, culture conditions and inhibitor treatments

*Chara australis* was grown in 10-50 l aquaria in a layer of soil, peat, and sand overlaid with distilled water, at a temperature of about 20°C and a 16/8h light/dark cycle provided by fluorescent lamps. Light intensity was low (about 5 μM.m^-2^.s^-1^ at the water surface) in order to prevent growth of epiphytes. Several weeks old, fully developed branchlets (side branches with limited growth) were clipped off from the 1st to the 3 rd whorl, and left in artificial fresh water (10^-4^ M NaCl, 10^-4^ M KCl, 10^-3^ M CaCl_2_, pH 5.6) until use. They generally consisted of one or two adjacent cells of 5-20 mm length.

Inhibitor stock solutions were: cytochalasin D (Sigma Aldrich, St. Louis, MO, USA; 10 mM in dimethyl sulfoxide (DMSO)), ikarugamycin (Santa Cruz Biotechnology, California, USA; 10 mM in DMSO), methyl -ß-cyclodextrin (Sigma Aldrich; 20 mM in distilled water), wortmannin (Enzo Life Sciences, Lausen, Switzerland; 5 mM in DMSO), brefeldin A (BFA; Sigma Aldrich; 70 mM in DMSO). Working solutions were prepared by dilution with artificial fresh water. The cells in control groups were treated with the equivalent amounts of DMSO. The pH of artificial fresh water was not significantly altered by addition of the inhibitors.

### *In vivo* staining with endocytosis markers and acidotropic dyes

As fluid-phase markers we used Alexa Fluor®488 hydrazide (Alexa 488 hydrazide, AF488HA), Alexa Fluor®568 hydrazide (Alexa 568 hydrazide, AF568HA) (both from Invitrogen, Carlsbad, CA, USA; 10  mM stock solution in distilled water) and Lucifer Yellow CH (Sigma Aldrich; 100 mM stock in distilled water). Working solutions were prepared by dilution with artificial fresh water. For *in vivo* staining of the plasma membrane, internodal cells were pulse-labeled for 5 min with the red fluorescent styryl dye FM4-64 (N-(3-triethylammoniumpropyl)-4-(6-(4-(diethylamino) phenyl)hexatrienyl)pyridinium dibromide; Invitrogen, Carlsbad, CA, USA) at a concentration of 10 μM diluted from a 500 μM stock solution in distilled water. Staining of acidic compartments was performed with 10 μM acidotropic dye Lysotracker Red DND-99 (Invitrogen; 1 mM stock solution in DMSO). The pulse-labelling experiments were performed at room temperature, unless otherwise stated.

### Confocal laser imaging, data collection and endocytosis quantification

The confocal imaging was performed on a Leica TCS SP5 laser scanning microscope coupled to an inverted microscope (Mannheim, Germany). For image acquisition a 63 x water immersion objective with a numerical aperture of 1.2 was used, and the pinhole size was set to 1 airy unit. For double-stained samples we always used the bidirectional, sequential scanning mode. The frame rate was typically 400 Hz at 512 x 512 image size, but occasionally, for cells with very high rates of cytoplasmic streaming, a frame rate of 700 Hz or 1400 Hz and smaller image size were necessary. For the excitation of AF488HA we used the 488 nm line of an argon laser and the emitted fluorescence was detected in the range 505–550 nm. Lucifer Yellow fluorescence was excited by the 405 nm line of a solid state laser and detected between 480 and 570 nm. The red fluorescent styryl dye FM4-64 was excited at 514 nm and the fluorescent signal was detected between 660 and 720 nm. AF568HA and Lysotracker Red were each excited with a diode pumped solid state laser at 561 nm and detected in the range 580–620 nm and 580-674 nm, respectively.

The *in vivo* detection and counting of stained endosomal structures in characean internodal cells is hampered by the strong autofluorescence of the compact chloroplast files. Therefore, we applied the “window technique”, which is generally used for light microscopy observation of the endoplasm in characean cells (Kamitsubo 1972). This method consists in strong local illumination of the cells, which results in chloroplasts bleaching, swelling and eventually detaching from the irradiated area. *Chara* cells used in our experiments were locally irradiated with the blue light of a halide microscope lamp for 3 min at least one day prior to the experiments. The resulting rectangular chloroplast-free window had a size of ca. 200 x 150 μm.

Local irradiation with high laser intensity (488 nm line of the Argon laser operated at 100 % for 1 min and guided through a 63x water immersion objective) was used to study the participation of fluorescently labeled organelles in wound healing. The irradiated area had size of about 40 x 40 μm. Laser irradiation at this wavelength, as also shown in studies on animal cells (Howard et al. 2011; Marg et al. 2012; Davenport et al. 2016), inflicted damage mainly on the plasma membrane whereas UV light, used for window formation, induces more extensive injury and chloroplast detachment.

Data were collected from series of individual time-lapse frames at least 1.3 s apart, showing fluorescent spots dispersed in the streaming cytoplasm. Images were processed with ImageJ (http://imagej.nih.gov/ij). For the quantitative analysis of endocytosis, the fluorescent particles in each frame and each channel were identified by eye and the number per area was manually counted (Choi et al. 2013; Chaparro-Garcia et al. 2015) using the Cell Counter plug-in. In order to obtain representative data sets, cumulated areas of at least 10000 μm^2^ were scored for each cell. Care was taken to analyze non-overlapping regions of the cytoplasm. The Pearson-Spearman correlation (PSC) plugin of ImageJ (French et al. 2008) was used for the co-localization analysis.

Further data processing, plots and statistical analysis were performed with Sigma Plot 13 (Systat Software, San Jose, USA). Differences between mean values were tested for statistical significance (*P* < 0.05) with the Mann-Whitney rank sum test or Student’s *t*-test.

### Immunofluorescence

The fixation and staining steps for indirect immunofluorescence used in this study are described in detail in Schmoelzer et al. (2011). As primary antibodies we used either rabbit polyclonal anti-OsSCAMP1 as *bona fide* TGN specific marker (Lam et al. 2007) generously provided by Liwen Jiang, University of Hong Kong, at a concentration of 40 μg per ml or a rabbit polyclonal anti-CaARA7 (GenScript; Piscataway, NJ, USA), as MVB marker at a concentration of 3.6 μg per ml (Hoepflinger et al. 2015). The secondary antibody was in both cases an anti-rabbit IgG Alexa Fluor**®**546 (Invitrogen) diluted 1:200.

### Electron microscopy

Cells processed for electron microscopy were treated with wortmannin (50 μM for 2 h or 25 μM for 30 min), BFA (200 μM for 30 min) or DMSO (1 % for 2 h, controls). Cells recovering for 2 h from a 30 min treatment with 25 μM wortmannin were also investigated.

Since mature internodal cells of *C. australis* are too large for high pressure freezing, chemical fixation was applied as described in Foissner (1991). Briefly, branchlet internodal cells were fixed for 20 min at room temperature in 1 % glutaraldehyde dissolved in phosphate buffer, pH 6.8. After several washes in buffer, cells were postfixed overnight at 4° C in 2 % OsO_4_ dissolved in buffer. Then the cells were dehydrated in an ethanol series at 4° C, and embedded in Agar low viscosity resin (Agar Scientific, Essex, Great Britain) via propylene oxide at room temperature.

Ultrathin sections were stained with uranyl acetate and lead citrate, and micrographs were taken at elastic bright-field mode with a LEO 912 transmission electron microscope equipped with in-column energy filter (Zeiss, Oberkochen, Germany).

## Results

### Time and concentration dependent internalization of FPE-markers in *Chara*

In order to study fluid-phase uptake in the multicellular green alga *Chara australis*, the fluorescent hydrazide dyes AF488HA, AF568HA and Lucifer Yellow were tested, and the optimal experimental conditions for their use were identified. Our experiments were performed with internodal cells of the branchlets which have a length of up to 2 cm and which are thus more convenient for life cell imaging than the up to 20 cm long internodal cells of the main axis.

**Fig. 1:**
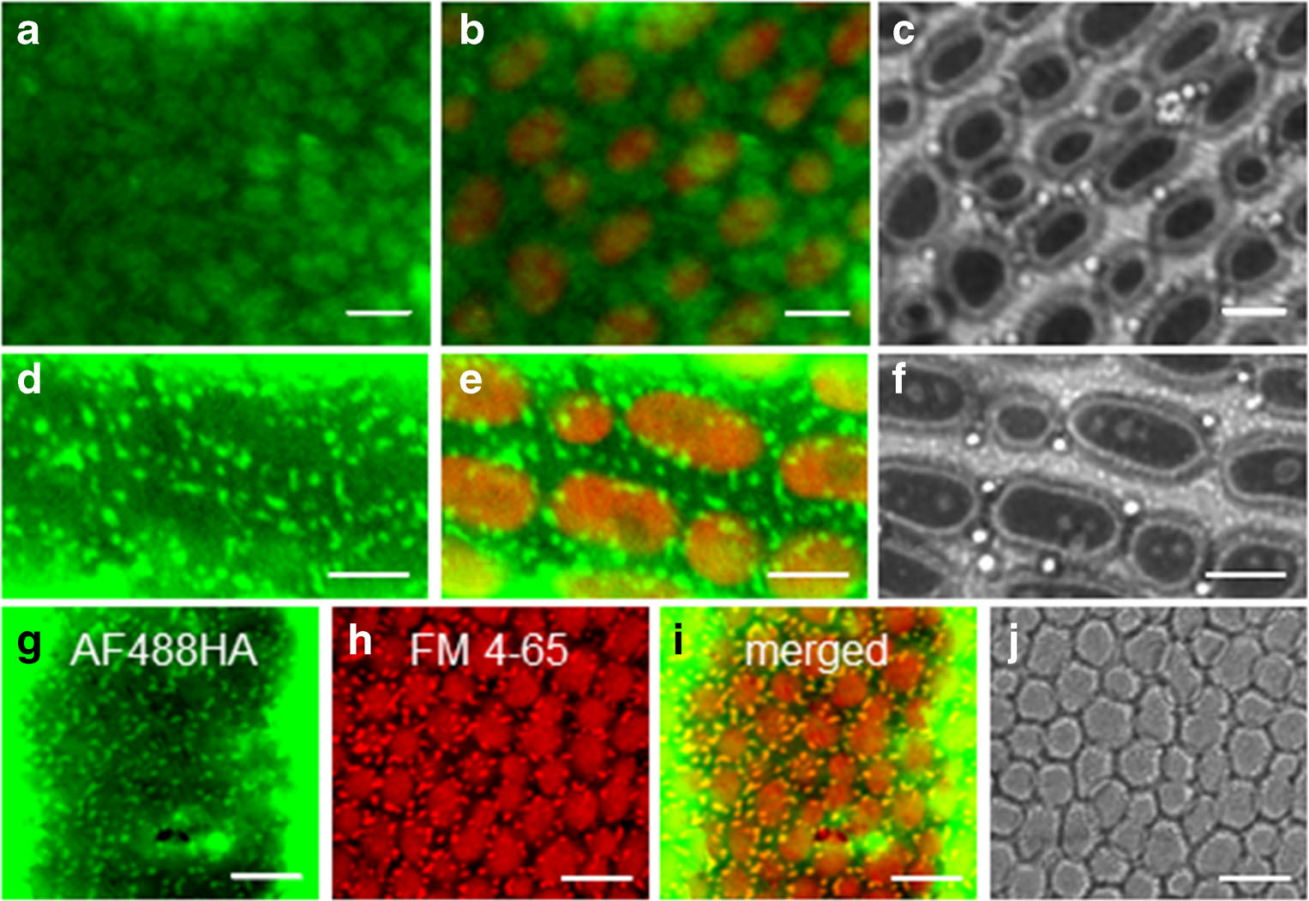
*Chara* cell wall and periplasmic space are stained by AF488HA. **a-c** Cloudy fluorescent pattern in the cell wall after 10 min-incubation with 4 mM AF488HA (**a**), AF488HA fluorescence merged with red autofluorescence of chloroplasts (**b**); (**c**) is the corresponding bright field image. **d-f** Punctate structures in the peripheral cytoplasm of a cell microscopied in 2 mM AF488HA (**d**). The AF488HA fluorescence is merged with autofluorescent chloroplasts in (**e**); the corresponding bright field image is shown in (**f**). **g-j** Double staining with 2 mM AF488HA (**g**) and the membrane-specific marker FM4-64 (**h**; 10 μM, 5 min pulse-staining); note also the red autofluorescence of the larger chloroplasts. The overlay (**i**) indicates that the structures stained by AF488HA correspond to the periplasmic space inside charasomes; (**j**) is the corresponding bright field image. Cells in (**d-j**) were imaged in AF488HA staining solution. Bars are 5 μm (**a-f**) and 10 μm (**g-j**)

*Chara* cells were incubated with AF488HA at concentrations between 10 μM and 4 mM, and dye uptake was followed in pulse-chase experiments, using different time combinations. After 10 min incubation with 4 mM AF488HA, conspicuous “cloudy” patterns became visible in the cell wall (Fig. 1a-c). When the cells were imaged in the staining solution (2 mM AF488HA), punctate or elongate immobile structures in the cortical cytoplasm (Fig. 1d-f) could be detected, which were similar in size and shape to the complex plasma membrane convolutions known as charasomes (Franceschi and Lucas 1980; Schmoelzer et al. 2011). Indeed, these structures co-labeled with the membrane-specific red fluorescent dye FM4-64 (Fig. 1g-j) indicating that AF488HA had access to and stained the extracellular (periplasmic) space of the charasomes. Staining of the periplasmic space disappeared after a 10 min washing step in dye-free medium, whereas residual staining of the cell wall persisted even after washout times up to 48 h.

The cytoplasm of the characean internodal cells comprises a stationary cortex with immobile chloroplasts arranged in parallel, helical files, and a motile phase (the endoplasm) which streams with high velocity (up to 100 μm s^-1^) (Video S1; Foissner and Wasteneys 2014). In the present study we were able to detect and analyze endocytic structures situated in the flowing endoplasm, beneath the chloroplast-free “windows” (see **Materials and Methods**), but the detection of AF488HA-containing vesicles in the cortical cytoplasm was impeded by the residual dye fluorescence in the cell wall.

The number of fluorescently labeled endoplasmic structures depended on AF488HA concentration, on the incubation time, and on temperature. The minimum dye concentration necessary for the detection of motile fluorescent spots after a 10 min pulse was 500 μM, in which case very few particles, hardly visible above the background began to appear after ca. 15 min. At the highest dye concentration used (4 mM), and the same pulse duration, the number of fluorescent spots was noticeably higher. However, the high background staining of the cell wall limited the detection and the quantification accuracy. Therefore, a 10 min pulse with 2 mM AH488HA, followed by 10 min washout in dye-free medium proved to be a good compromise, that enabled a good detectability of endocytic compartments, while minimizing the disturbing fluorescence of the cell wall, and was used in most of the experiments presented here. In intact guard cells of *Vicia faba* the lowest AF488HA concentration that allowed *in situ* observation of endocytic vesicles was 4 mM (Gall et al. 2010), which is 8 times higher than the concentration limit found by us. A likely explanation lies in the different cell-specific turgor pressures, since fluid-phase endocytosis must always overcome this barrier. The cells of open stomata have the highest known turgor, with values up to 4.5 MPa (Meckel et al. 2004), whereas in *Chara* cells the value is considerably lower (0.6 MPa; Shepherd et al. 2001).

**Fig. 2:**
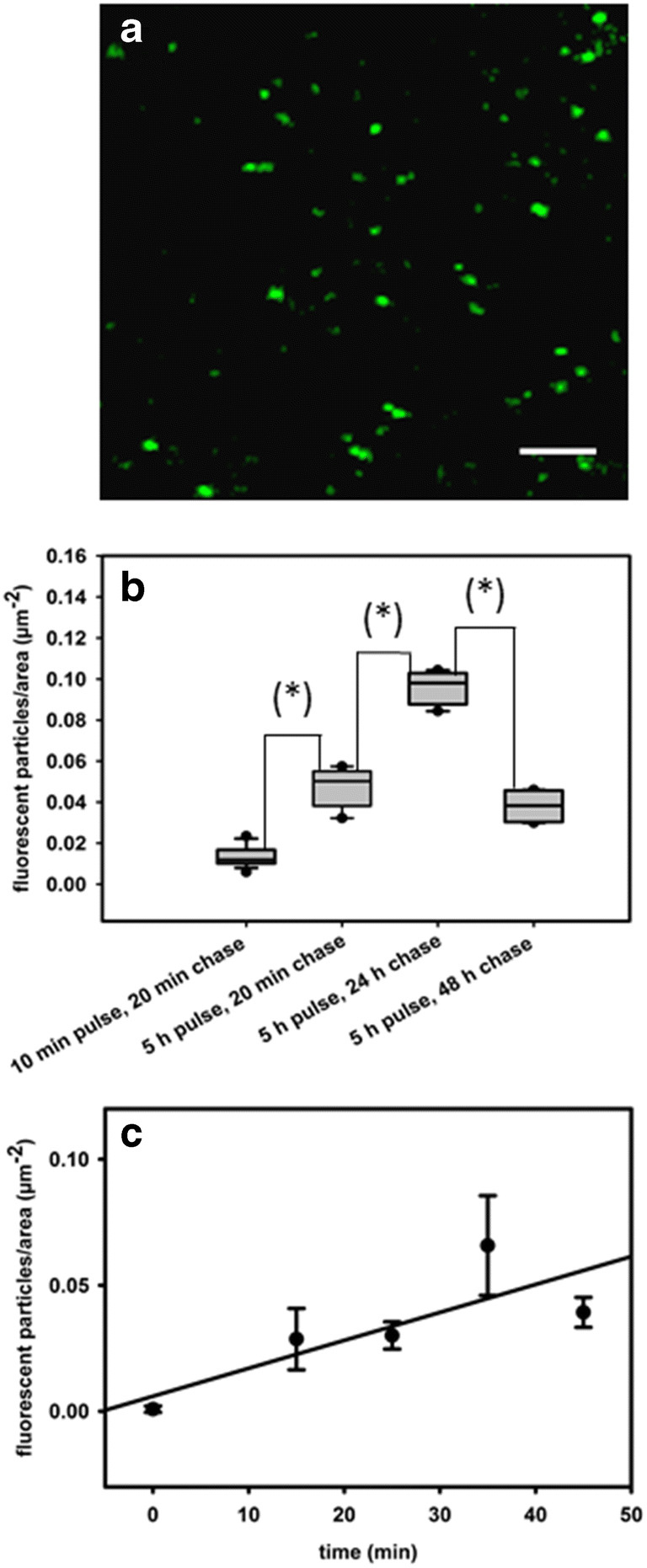
Uptake of the fluid phase marker AF488HA in *Chara* cells shows linear time dependence and is inhibited by cold. **a** After 10 min incubation with 2 mM AF488HA and 20 min chase, green fluorescent punctate structures are visible in the cytoplasm. Bar is 5 μm. **b** Box plot of the number of fluorescent particles per area in relation to different pulsing and chasing times, using a dye concentration of 2 mM. The number of AF488HA-positive structures clearly increased after longer incubation times (5 h versus 10 min) and decreased down to initial levels after 48 h. The asterisks indicate significant differences between the data groups connected by lines (t-test, P<0.01). **c** Representative time-course plot of AF488HA uptake. *Chara* cells (n = 6) were pulsed for 45 min with 2 mM AF488HA at 4°C, and then incubated for different time periods in dye-free medium at room temperature. At time t = 0 no particulate staining could be detected in the streaming cytoplasm, indicating the lack of internalization at 4°C. At room temperature, the fluid phase internalization increased linearly with time

When we chased the marker 10 to 20 min after pulsing, fluorescent structures with sizes from 282 nm to 1100 nm (average diameter 496 ± 16 nm) appeared in the flowing cytoplasm (Fig. 2a). These values are rough size estimates, which might not reflect accurately the real endosome dimensions, since we cannot exclude that small particle aggregates that moved with the same velocity were seen as individual endosomes. The number of fluorescent mobile structures per μm^2^, used here as a parameter to quantify fluid-phase endocytosis, increased significantly with longer incubation times (see abundant fluorescent organelles in Video S2 after 5 h incubation) and longer chasing times (Fig. 2b, c). The increase observed after a chasing time of 24 h was surprising. We assume that a continuous, slow accumulation of AF488HA might have taken place in some endocytic compartments, that gradually received the dye by repeatedly fusing with small and/or insufficiently labeled and thus invisible vesicles. In addition, the cell wall space might have served as a dye reservoir over the long term.

Whereas some reports on fluid-phase endocytosis in plant cells identified the vacuole as the final destination organelle (Oparka and Prior 1988; Robinson and Milliner 1990; Oparka 1991; Etxeberria et al. 2005), others did not find the external fluid marker in this compartment (Baluska et al. 2004). We followed the distribution of the internalized dye over two days, but we could detect AH488HA fluorescence neither in the central vacuole, nor in smaller vacuoles located in the cytoplasm. Intriguingly, bright fluorescent particles were still present in the streaming endoplasm up to 48 h after the pulse-staining (Fig. 2b), which suggests that the fluid-phase marker was sequestered within compartments with a slow turnover rate. We further examined the internalization of the AH488HA under low temperature conditions in order to test whether the uptake could be inhibited by cold. The cells (n=7) were preconditioned by incubation at 4°C for 10 min before dye addition, then exposed to 2 mM AF488HA for 45 min on ice, and subsequently suspended in ice-cold dye-free medium. Immediate inspection under the confocal microscope revealed no visible particulate staining of the cytoplasm, which demonstrated that uptake of fluid-phase is an active, energy using process, in accordance with previous reports (Etxeberria et al. 2007a, 2012; Onelli et al. 2008). The low temperature inhibition proved to be reversible, as the number of fluorescent particles in the cytoplasm increased linearly with time during adaptation to room temperature (Fig. 2c).

**Fig. 3:**
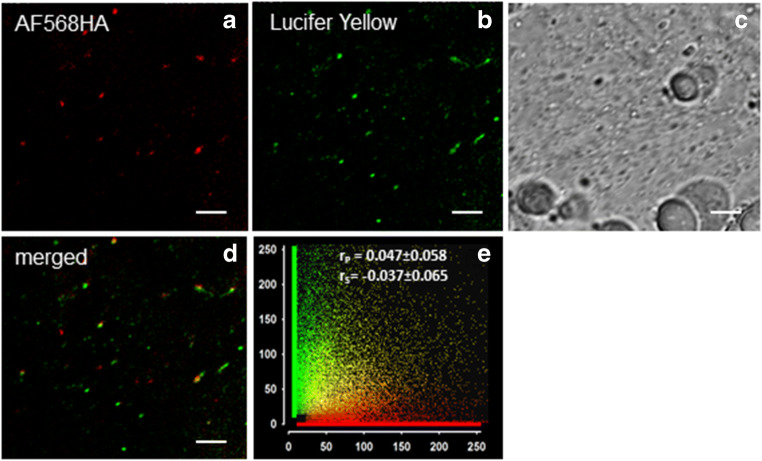
Distribution of sequentially internalized fluid phase markers. *Chara* cells were pulse-labeled for 30 min with 2 mM AF568HA, washed in dye-free medium and then incubated for 30 min with 10 mM Lucifer Yellow. Images were taken after 10 min wash in dye-free medium. The punctate structures indicate endocytic compartments stained with: (**a**) AF568HA, (**b**) Lucifer Yellow; (**c**) corresponding bright field image; (**d**) merged image of (**a**) and (**b**). **e** Scatterplot derived from the co-localization analysis of AF568HA positive and Lucifer Yellow stained particles. The quantification was performed using the PSC plug-in of ImageJ (French et al. 2008) on 627 individual fluorescent spots manually masked from time-lapse recordings taken from 3 different cells. Pearson’s (r_p_) and Spearman’s correlation (r_s_) coefficients are given as means±SD. Bars in (**a-d**) are 5μm

We next tested the red fluorescent fluid-phase marker AH568HA. It has the same net charge as AH488HA, a similar molecular brightness (60.72 mM^-1^cm^-1^, as compared to 67.16 mM^-1^cm^-1^ for AH488HA; values calculated as molar extinction coefficient **∙** quantum yield using data from www.invitrogen.com), but a higher molecular weight (MW_AF568HA_=730.74 vs. MW_AF488HA_=570.48) and consequently a larger Stokes radius. Under incubation conditions established to be optimal for AF488HA (2 mM and 10 min pulse), very few fluorescent structures were visible in the cytoplasm of *Chara* cells when using AH568HA. In order to detect a high number of punctate bright fluorescent structures in the endoplasm (Fig. 3a), the incubation time had to be extended to 30 min.

We further tried the very popular fluid-phase marker Lucifer Yellow, which has a rather dim fluorescence (5 mM^-1^cm^-1^ brightness, calculated with data from Stewart (1981)), and a slightly smaller Stokes radius than AF488HA (Heyman and Burt 2008). After pulsing for 10 min with 10 mM Lucifer Yellow, abundant fluorescent particles were visible in the endoplasm of *Chara* cells (Fig. 3b). Lower concentrations gave unsatisfactory staining, most probably because of the lower molecular brightness of the dye.

No toxic effects could be observed for the fluorescent markers tested at these concentrations. During the course of the experiments, all cells displayed normal cytoplasmic streaming, and the labeled cells had a survival rate of 100 %.

### Tracing the spatio-temporal pathway of internalized AF488HA

During initial steps of endocytosis, primary endocytic vesicles internalize membrane and external fluid-phase cargo. Endocytosed material is then progressively delivered to early endosomes through several membrane fusion and content mixing steps, which occur in a well-regulated spatio-temporal sequence. These events are well documented in animal cells (Rink et al. 2005), but comparatively little is known to date about these processes in plant cells.

In order to get insight into endosome progression we performed sequential pulse-staining with different fluorescent dyes. *Chara* cells were first incubated with 2 mM AF568HA for 30 min, which allowed extensive distribution of the fluid-phase marker to compartments of the endocytic pathway. After a 10 min washing step, the cells were incubated for 30 min with 10 mM Lucifer Yellow, and after another 10 min wash in dye free medium the cells were examined with the confocal microscope. As illustrated in Fig. 3d, very few endosomes were stained by both markers, resulting in a poor co-localization of the fluorescent signals (Fig. 3e). This indicates that the two dyes were entrapped in distinct organelles, and accordingly, no significant fusion with content mixing occurred between compartments recently labeled by Lucifer Yellow, and earlier endocytic structures stained by AH568HA. Thus, the emerging picture is that fluid phase cargos loaded consecutively at a time interval of ca. 80 min did not “meet” within the same vesicular structures, but were spatially sorted into different late compartments, with diminished fusion competence. These results are similar to findings reported by (Salzman and Maxfield 1988, 1989), who showed that in mammalian cells, fusion occurred mainly between primary endocytic vesicles formed at 5 to 15 min interval, and at the early endosome, whereas fusion competence decayed for late endosomes, situated downstream on the endocytic pathway.

**Fig. 4:**
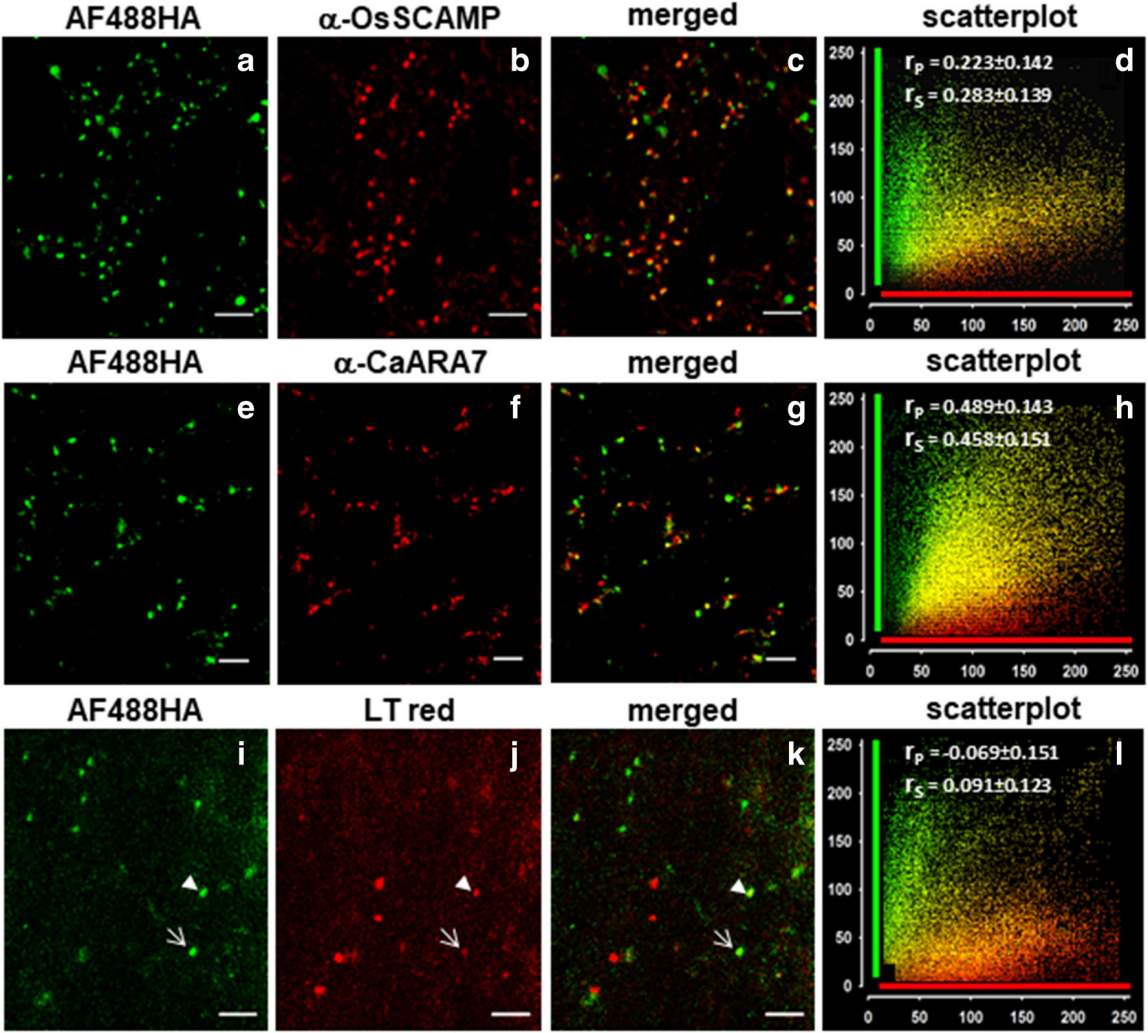
AF488HA distributes to early and late compartments of the endocytic pathway. **a-d** Immunofluorescence of AF488HA stained cells (**a**) with anti-OsSCAMP, a TGN-specific antibody (**b**). Only few organelles are stained by both markers, as shown in the merged image (**c**) and in the scatterplot (**d**). **e-h** Immunofluorescence of AF488HA stained cells (**e**) with anti-CaARA7 (**f**), which specifically recognizes MVBs. Merged image (**g**) and scatterplot (**h**) reveal a higher degree of co-localization. **i-l** Organelles stained by fluid phase marker AF488HA (**i**) are rarely stained by the acidotropic dye Lysotracker Red (**j-l**). The arrowheads in (**i-k**) point to an acidic compartment intensely stained by the fluid phase marker, whereas the arrows identify a less acidic endosome (weak fluorescence in the red channel) that is strongly stained by AF488HA. Cells were incubated with 2 mM AF488HA for 30 min prior to fixation for immunofluorescence (**a** and **e**). For the comparison with the acidotropic dye cells were pulse-labeled for 10 min with AF488HA (**i**) and then incubated with 5 μM Lysotracker red for 30 min. Consequently, AF488HA was chased in the endocytic compartments after times comparable to the immunostaining experiments. For the co-localization analysis the PSC plugin of ImageJ was used; 230 to 526 green fluorescent structures from 3 cells were manually masked and the resulting cumulative scatterplots together with the Pearson’s (r_P_) and Spearman’s (r_S_) correlation coefficients are shown in (**d**), (**h**) and (**l**). Bars are 5 μm

We further performed immunofluorescence microscopy and used antibodies to label early (TGN) and late compartments, i. e. MVBs and prevacuolar compartments (PVCs). In order to prove if after 30 min exposure to 2 mM AH488HA the marker is present in early endocytic compartments, we immuno-labeled the cells with OsSCAMP, an antibody used as a *bona fide* marker for TGN (Lam et al. 2007). As illustrated in Fig. 4a-c, the fluorescent hydrazide, which is readily fixable by glutaraldehyde, stained the lumen of numerous endosomes, some of them bearing epitopes recognized by anti-OsSCAMP. However, the co-localization of the two markers is low (Fig. 4d), indicating that after 30 min the internalized fluid-phase can only occasionally be found in early endocytic compartments (TGNs). It is thus probable that, during this time interval, the fluid-phase cargo distributed predominantly to organelles situated downstream the TGN, or on a TGN subpopulation that lacked the SCAMP epitopes.

ARA7 is a member of the RAB5 GTPases group of higher plants, which localizes to late endosomes (Lee et al. 2004; Ueda et al. 2004), and an ARA7 homolog, CaARA7 was identified in *Chara australis* (Hoepflinger et al. 2015). Here we used an affinity-purified antibody against CaARA7 in order to test if external fluid-phase cargo can be identified in late compartments after 30 min incubation with the dye. Figure 4e-h illustrates that the endocytic compartments labeled by the FPE marker and the CaARA7-positive endosomes match to a higher degree, in support of the hypothesis that after 30 min incubation much of the dye reached late endocytic compartments.

In a further attempt to identify the membrane-bound compartments that enclosed internalized fluid-phase marker, we used the acidotropic dye Lysotracker Red. After a 10 min pulse with 2 mM AF488HA, the cells were incubated for 30 min in 5 μM Lysotracker Red. Confocal imaging of the cells revealed poor co-localization of the two fluorescent signals (Fig. 4i-l), suggesting that after at least 40 min (pulse time included) the fluid-phase marker can be identified mainly in neutral, and much less in acidic endosomes. In plant cells, unlike in animal cells, H^+^-ATPase activity was detected in the early endosomes which consequently have an acidic lumen, but is lacking in the late endocytic compartments (MVBs/PVC) which have an almost neutral internal pH (Dettmer et al. 2006; Martiniere et al. 2013; Robinson and Pimpl 2014; Luo et al. 2015; Robinson and Neuhaus 2016). This finding, therefore, reinforces the idea that after ca. 30 min, the internalized fluid-phase marker passed from early to late endocytic compartments.

### Fluid-phase - and membrane endocytosis markers co-localize following concomitant pulse incubation

**Fig. 5:**
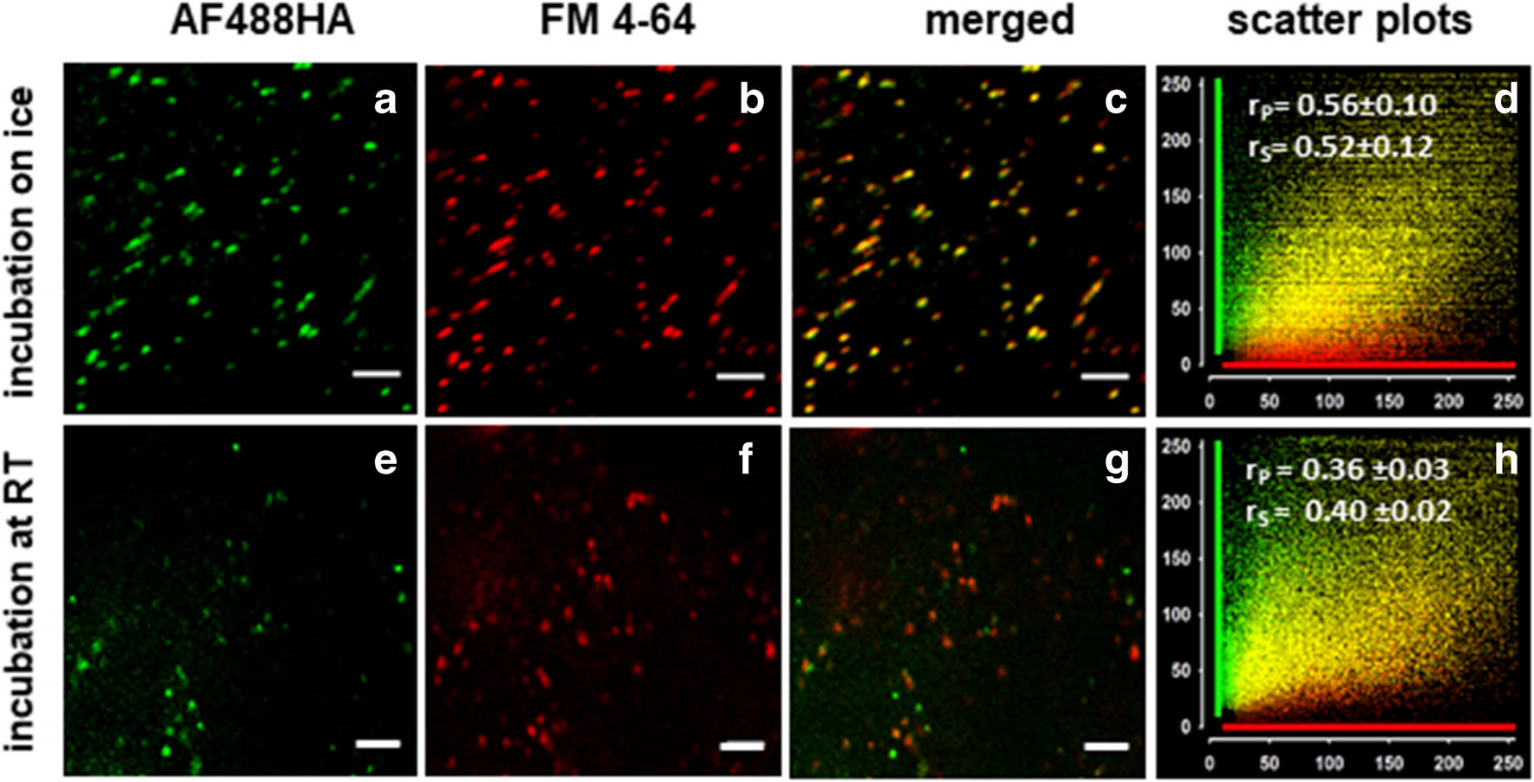
Co-localization of fluorescent fluid-phase and membrane markers after simultaneous pulse-staining. *Chara* cells were simultaneously incubated with 2 mM AF488HA and 10 μM styryl dye FM4-64 for 45 min on ice (**a-d**) or 10 min at room temperature (**e-f**) and chased after 20 to 40 min. The scatterplots show that the fluorescent signals in the endocytic compartments largely co-localize, proving the endocytic nature of fluid-phase uptake. Bars are 5 μm. For scatterplot (**d**) 1000 green fluorescent structures from 3 cells and for scatterplot (**h**) 1473 structures from 14 cells were analyzed with the PSC plugin of ImageJ. Pearson’s (r_P_) and Spearman’s (r_S_) correlation coefficients are indicated

As an additional proof of the endocytic nature of fluid-phase uptake in *Chara* we used the styryl fluorescent membrane marker FM4-64 in conjunction with the FPE marker AF488HA. Previous work has shown that styryl dyes stained the plasma membrane of *Chara* internodal cells, including convoluted domains (charasomes) if present (Klima and Foissner 2008). Few minutes after addition, these dyes were found to label punctate organelles in the stationary cortex and in the streaming endoplasm. In the present work, we pulsed the cells concomitantly with 2 mM AH488HA (green fluorescence) and 10 μM FM4-64 (red fluorescence) on ice and chased the two markers after 40 min at room temperature. As can be seen in Fig. 5a and b, many mobile, punctate fluorescently labeled structures can be identified in the endoplasm, and the overlay in Fig. 5c validates the endocytic nature of the fluid-phase uptake. Co-localization analysis (French et al. 2008) revealed good co-localization of the fluorescent signals (Pearson’s r_s_ = 0.566 ± 0.106, Spearman’s r_p_ = 0.524 ± 0.121, Fig. 5d). The values of the correlation coefficients generally reflect the relationship between the intensities of the fluorophores not only their distribution to the same compartments. Another possibility to describe the distribution pattern of two fluorescent markers is to quantify the frequency of the co-occurrence in the same endocytic compartments, irrespective of the signal intensity. Measurements using the Cell Counter plugin of ImageJ revealed that 94.2 % of the green fluorescent particles displayed also red fluorescence (1000 particles from 3 cells were analyzed).

When we used short pulse incubations for the parallel uptake of AF488HA and FM-dyes at room temperature, and the markers were chased after 20 to 30 min, we found that: (a) the number of endocytic structures stained by the membrane styryl dye exceeded considerably the number of AH488HA-positive ones, and (b) the membrane of more than 40 % of the AF488HA-enclosing endosomes was not stained by FM4-64 (Figs. 5e-f). The lack of full co-localization between FM dyes and other endocytic markers is not restricted to *Chara* cells, but is a frequently reported phenomenon (Chow et al. 2008; Etxeberria et al. 2009; Gall et al. 2010; Bandmann et al. 2012; Li et al. 2012; Hoepflinger et al. 2013; Palocci et al. 2017). Although we cannot exclude selective cargo sorting, we hypothesize that the lack of full co-localization of fluid-phase and endocytic membrane markers at room temperature reflects a staining and/or detection problem probably due to different retention times within the cell wall and hence different arrival of markers at the plasma membrane. This finding also points to the limits of using these endocytosis markers and the need for careful experiments.

### The compartments that internalize AF488HA are wortmannin- and BFA-sensitive

**Fig. 6:**
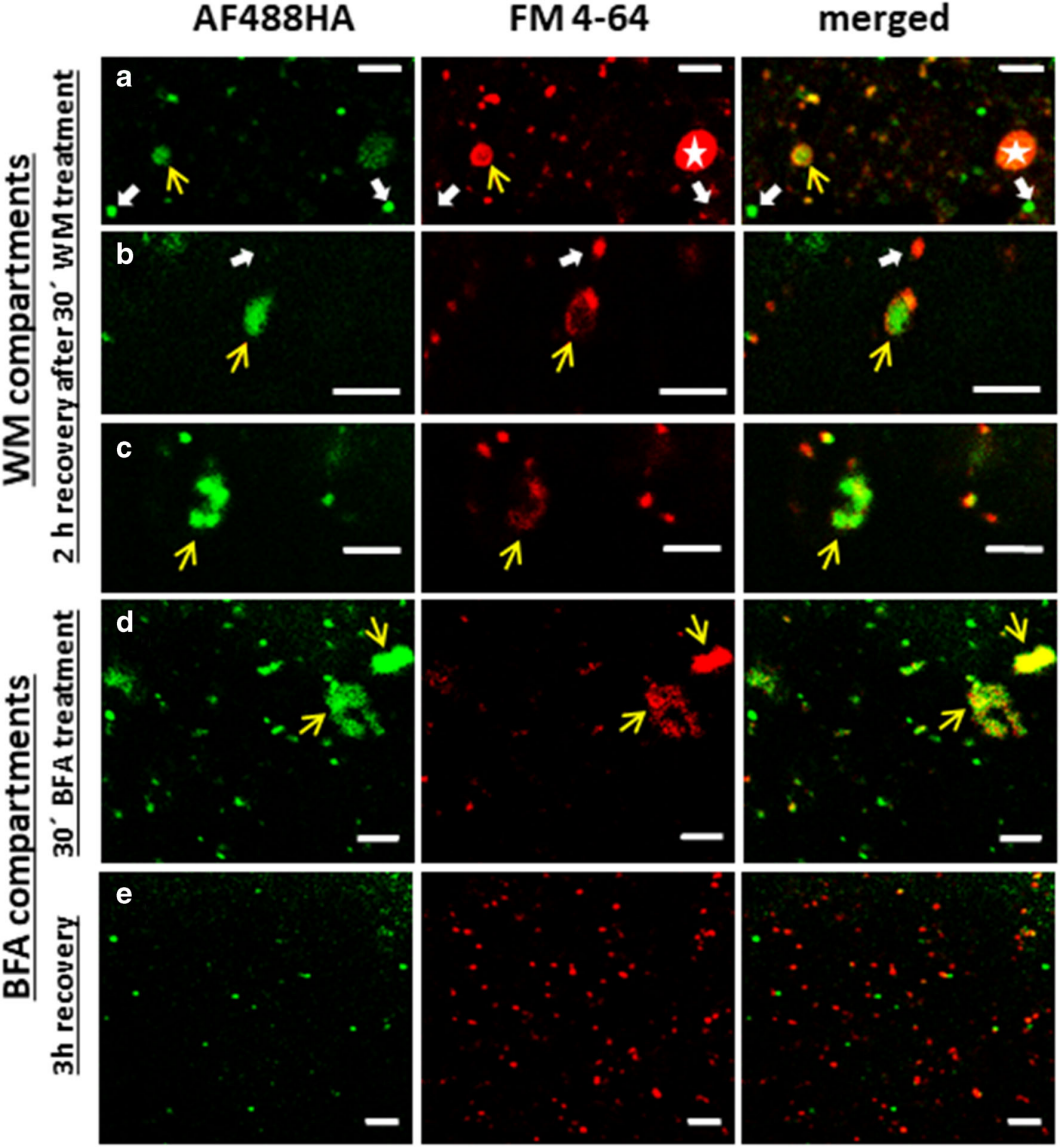
Localization of plasma membrane- and fluid phase markers in wortmannin- and BFA-treated *Chara* cells. Cells were simultaneously pulse-labeled with 2 mM AF488HA and 10 μM FM4-64 for 10 min before adding the inhibitors. **a-c** Three examples for endoplasmic organelles in cells “recovering” for 2 h from 30 min treatment with 25 μM wortmannin (WM). Thin yellow arrows indicate compartments labeled by both dyes and thick white arrows indicate single-stained compartments. The white star in (**a**) marks a red fluorescent chloroplast. **d** and **e** Endoplasmic organelles in cells treated with 200 μM BFA for 30 min (**d**; arrows indicate typical organelle-aggregates known as BFA-compartments) and in cells recovering for 3 h from BFA-treatment (**e**). Bars = 5 μm

**Fig. 7:**
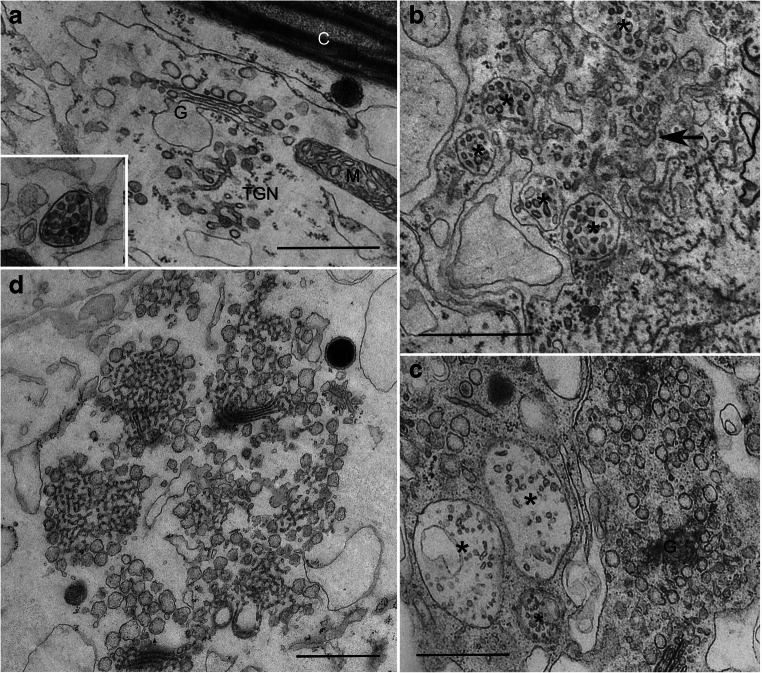
Effect of wortmannin and BFA on the fine structure of internodal cells. **a** Golgi body (G), TGN and MVB (inset) in the endoplasm of a control cell. M = mitochondrion, C = chloroplast. **b** Formation of aggregates consisting of TGN-tubules (arrow) and MVBs (asterisks) in a cell treated with 50 μM wortmannin for 2 h. **c** Huge MVBs (asterisks) in a cell recovering from wortmannin treatment (25 μM wortmannin for 30 min, 2 h recovery in artificial fresh water). **d** Agglomeration of Golgi bodies and TGN following 30 min treatment with 200 μM BFA. Bars are 1 μm

Wortmannin is a fungal metabolite that inhibits phosphatidylinositol-3 (PI3) and phosphatidylinositol-4 (PI4) kinases and is widely used as an inhibitor of endocytosis. In *Chara* cells, we found that wortmannin did not inhibit endocytosis up to a concentration of 50 μM, but induced the formation of “mixed compartments” consisting of MVBs and membranous material probably derived from TGN, as well as enlargement of MVBs by homotypic fusions, especially in cells recovering from wortmannin treatment (Foissner et al. 2016). In the present study, we examined the staining pattern of wortmannin-induced compartments using fluid-phase and membrane markers. For this, *Chara* internodal cells were simultaneously pulse-labeled with 2 mM AF488HA and 10 μM FM4-64 for 10 min at room temperature, and then treated with 25 μM wortmannin for 30 min. Immediate examination of the cells showed largely the same staining patterns as controls, but inspection after 2 h revealed the appearance of large membrane-bound compartments that were labeled by both fluid-phase and membrane markers (Fig. 6a-c; yellow arrows). Occasionally, larger endosomes with a size of about 1.5 μm were noticed, that presented only one type of fluorescence (white thick arrows in Fig. 6a and b), and probably originated from homotypic fusions of single-stained compartments. The fine structure of control cells and the enlarged wortmannin compartments is presented in Fig. 7. It proves that they are of MVB origin and remnants of the TGN. The presence of both fluorescent markers in these large wortmannin compartments is consistent with the immunofluorescence data and suggests that after longer chasing times the membrane and fluid phase cargo reached late endocytic compartments.

We next investigated the effect of the fungal toxin BFA, a popular inhibitor of protein trafficking, on constitutive endocytosis in *Chara*. For *in vivo* staining, *Chara* cells were simultaneously pulse-labeled with 2 mM AF488HA and 10 μM FM4-64 for 10 min at room temperature, then treated with 200 μM BFA for 30 min. In BFA-treated cells, very large organelle-aggregates were frequently detected in the flowing cytoplasm. Their endocytic origin was demonstrated by the presence of both fluid-phase and membrane markers AF488HA and FM4-64, respectively (Fig. 6d). As shown in the scatter plot of Fig. S1, the fluorescent signals in the green and red channel revealed a high degree of co-localization in BFA-compartments. Enlarged single-stained compartments were not detected. After 3h incubation in dye-free medium the cells recovered and regained the fluorescent punctate staining of the cytoplasm, demonstrating that the BFA effect is readily reversible (Fig. 6e), in accordance with findings by Geldner et al. (2001), Tse et al. (2006) and Lam et al. (2009). As with wortmannin, we examined the fine structure of the endocytic compartments after BFA treatment. Electron microscopy revealed typical BFA compartments consisting of Golgi and TGN/vesicle aggregates (Fig. 7d), as described from other plant cells (e.g. Satiat-Jeunemaitre and Hawes 1992; Hause et al. 2006; Lam et al. 2009; Stierhof et al. 2013). These observations together with the fluorescence microscopy data suggest that during the 10 min pulse the dyes stained extensively the early endocytic compartments, and the rapid action of BFA concentrated the markers in TGN-containing BFA-compartments. Notably, qualitative inspection of the punctate structures in the red and green fluorescence channel revealed that the co-localization of two endocytosis markers diminished after the recovery phase (not shown).

### Uptake of AF488HA is not disturbed by the actin depolymerizing agent cytochalasin D but is inhibited by ikarugamycin and methyl-ß-cyclodextrin

Next, we investigated the effect of several inhibitors on the internalization of AH488HA in order to find out whether fluid phase endocytosis in *Chara* cells depends on actin and clathrin.

In the inner cortex cells of maize root apices, the uptake of Lucifer Yellow was found to be dependent on F-actin (Baluska et al. 2004). In order to investigate if actin is similarly involved in the fluid-phase uptake and internalization in *Chara*, we used cytochalasin D which reversibly inhibits actin-based motility and transiently reorganizes the delicate cortical actin filament meshwork in characean algae into short, thick rods (Foissner and Wasteneys 2000; Foissner and Wasteneys 2007)*.* Internodal cells were treated with 10, 50 or 100 μM cytochalasin D for 30 min, and then stained for 10 min with 2 mM AF488HA containing the corresponding inhibitor concentrations. Even at 100 μM, which completely arrested cytoplasmic streaming, we were able to detect numerous AF488HA-stained particulate structures, which performed wiggling, probably saltatory movements in the stagnant endoplasm (Fig. S2, Video S3).

**Fig. 8:**
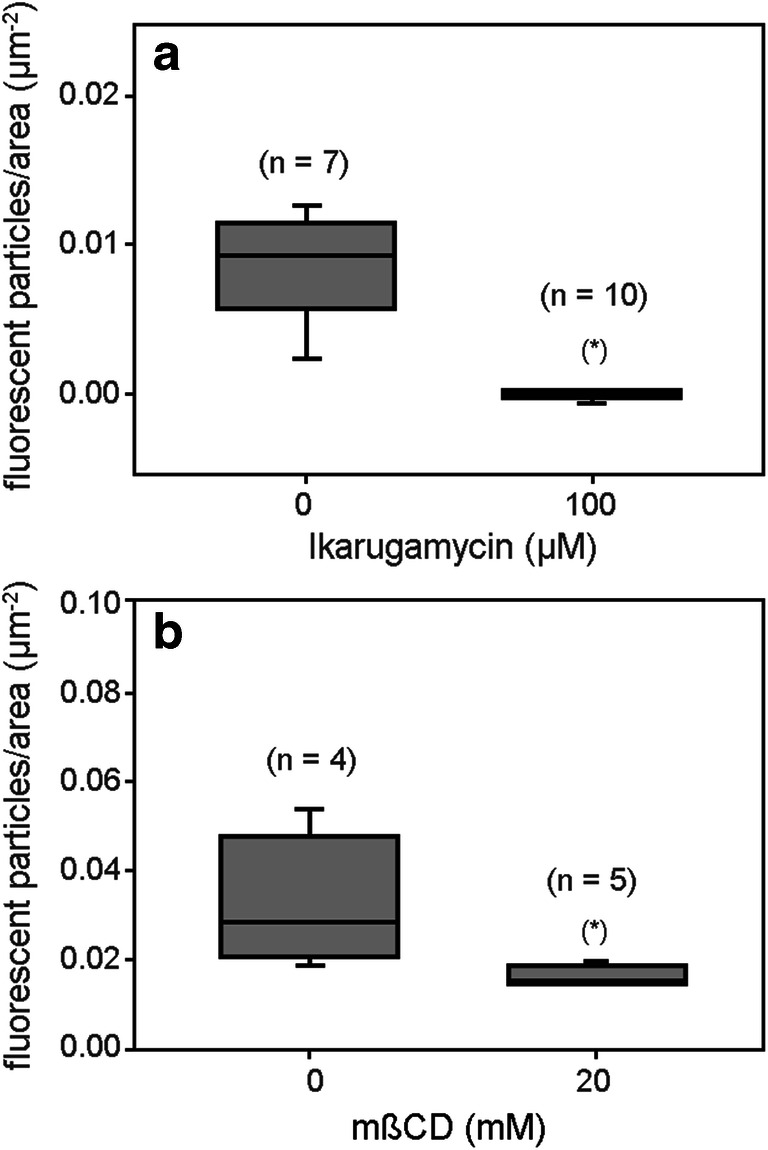
Constitutive FPE in *Chara* is sensitive towards ikarugamycin, an inhibitor of clathrin-dependent endocytosis, and methyl-ß-cyclodextrin, an inhibitor of sterol-dependent endocytosis. Internodal cells were incubated for 30 min with the respective inhibitors or mock- treated with artificial fresh water with appropriate additions of DMSO at room temperature. The cells were subsequently pulse-stained for 10 min with 2 mM AF488HA, washed for 10 min in artificial fresh water and then examined under the confocal microscope. **a** Ikarugamycin significantly inhibited fluid phase uptake at a concentration of 100 μM (P <0.01). **b** 20 mM methyl-ß-cyclodextrin (mßCD) also significantly reduced the number of particulate fluorescent structures in the cytoplasm (P = 0.026). The Mann-Whitney rank sum test was applied for the comparison between control and treated groups and one-tailed P values were determined. The numbers in brackets indicate the number of cells used in the experiments and the asterisks mark significant differences between treated and control cells

We further used the pharmacological tools ikarugamycin and methyl-ß-cyclodextrin, known to inhibit the formation and the trafficking of endocytic vesicles. Ikarugamycin is an antibiotic agent considered to specifically inhibit clathrin-dependent endocytosis in animal and plant cells (Hasumi et al. 1992; Luo et al. 2001; Moscatelli et al. 2007; Onelli et al. 2008; Bandmann et al. 2012; Elkin et al. 2016). Methyl-ß-cyclodextrin is a sterol-complexing agent that disrupts cholesterol-rich membrane domains and was shown to interfere with sterol-dependent endocytic pathways in plant cells (Rodal et al. 1999; Grebe et al. 2003; Boutte and Grebe 2009). We recently reported that in *Chara* internodal cells, ikarugamycin did not inhibit clathrin-coat formation, but affected the release of coated vesicles from the plasma membrane at sub-micromolar concentrations. At concentrations of at least 100 μM, ikarugamycin significantly inhibited the constitutive internalization of FM dyes (Hoepflinger et al. 2017). We also showed that 20 mM methyl-ß-cyclodextrin markedly repressed the uptake of a fluorescent membrane marker (Hoepflinger et al. 2017). In the present work, we found a similar effect of ikarugamycin and methyl-ß-cyclodextrin on the uptake of the fluid-phase marker AF488HA. Both inhibitors (100 μM ikarugamycin or 20 mM methyl-ß-cyclodextrin) significantly reduced the relative number of fluorescently labeled compartments in the endoplasm as shown in Fig. 8a and b.

### Fluid-phase markers accumulate in compartments involved in wound healing

**Fig. 9:**
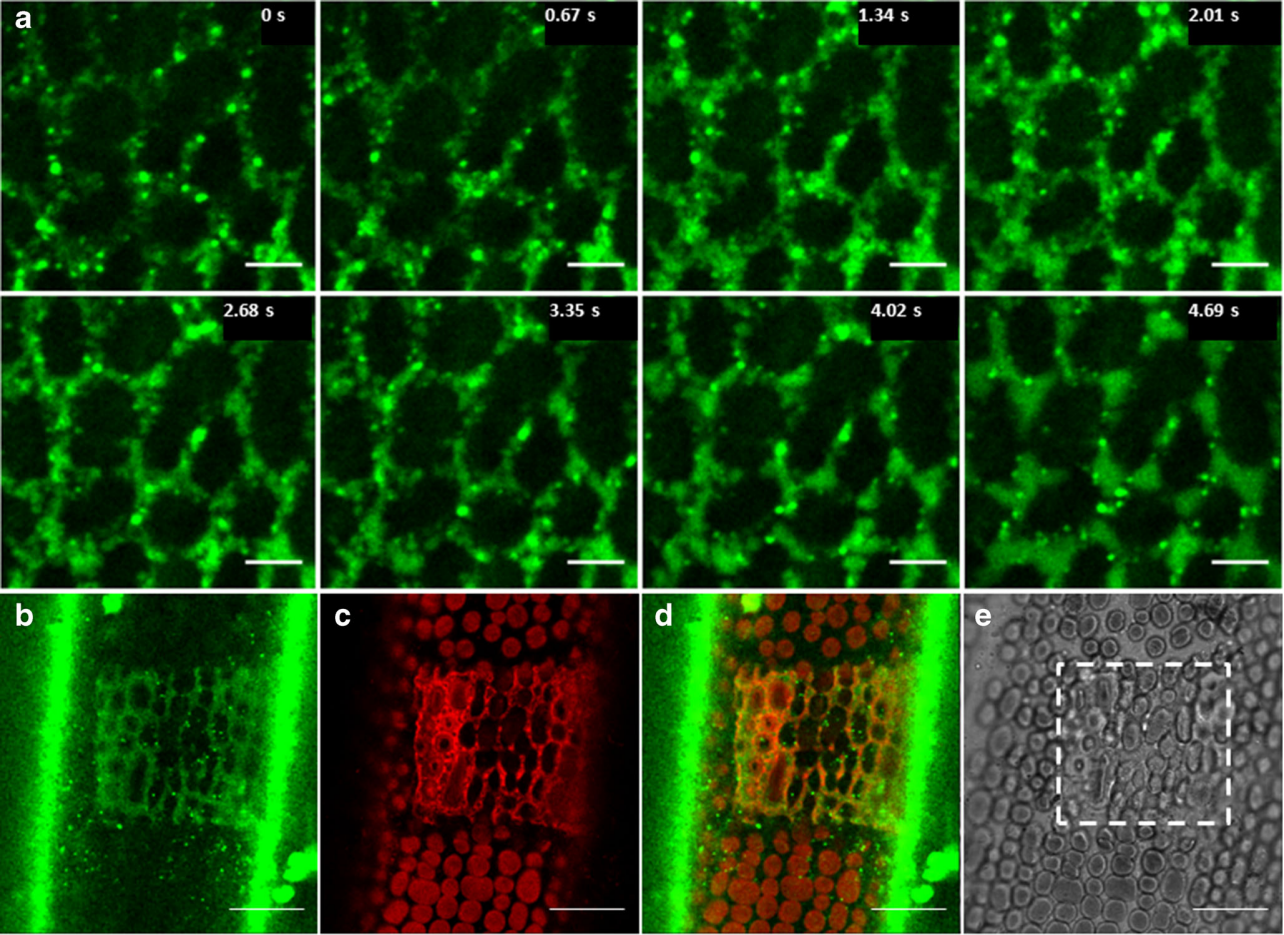
Plasma membrane repair after laser injury involves participation of compartments stained by fluid phase markers and plasma membrane dyes. *Chara* cells were first stained with 2 mM AF488HA for 5 h, then pulsed with 10 μM FM4-64 for 10 min, and finally washed and suspended in dye-free artificial fresh water for confocal microscopy. Cells were injured by irradiating a region of 41 x 41 μm (the focal plane was slightly above the bleached and detaching chloroplasts) with full power of the 488 nm line of the Ar laser operated at 1.2 mW. **a** Sequential images from a 4.69 s long time series, illustrating the on-going accumulation of vesicles loaded with fluid phase marker at the wound site. **b-e** Wound region of a cell, which was stained with AF488HA and FM4-64 as described above and injured after a 2 d wash in AFW. The deposited material contains fluid phase marker (**b**) and membranes stained by FM4-64 (**c**), both indicating the participation of long-lived endocytic compartments in wound repair. **d** is the overlay of the fluorescent signals in (**b**) and (**c**), and **e** is the corresponding bright-field image. The rectangle **e** in indicates the border of the wound. Note that the green vertical stripes in (**b**) represent AF488HA-residual fluorescence of the cell wall (the longitudinal axis of the cell is parallel to the vertical sides of the image). Bars are 5 μm (**a**) and 20 μm (**b-e**)

In *Chara* internodal cells, plasma membrane wounding can be achieved by mechanical injury, by local irradiation of the cell with intense UV or blue light. We have previously shown that plasma membrane repair following local injury of internodal cells involves not only exocytosis of secretory vesicles, but also deposition of putative recycling endosomes, probably derived from the TGN (Klima and Foissner 2008; Foissner and Wasteneys 2012). We now investigated the involvement of fluid-phase containing organelles in the process of membrane repair and wound healing. In order to extensively label endocytic compartments far downstream the early endosome (e.g. putative recycling endosomes), the cells were incubated with 2 mM AH488HA for 5h. They were then pulsed for 10 min with 10 μM FM4-64 and transferred into dye-free medium. Local injury was performed with the 488 nm line of the Argon laser operated at 100 % intensity for 1 min, and using a 63x water immersion objective. Within seconds, an impressive accumulation of AF488HA stained particles was observed at the wounded area. They docked to the injured site (Video S4), and eventually delivered their content to the periplasmic space (Fig. 9a and b, Video S4). The presence of the fluorescent membrane marker within the secreted material (Fig. 9c and d) indicated that also FM4-64-positive endosomes gathered at the wound site, obviously participating in the membrane repair and wound-healing process, as formerly described (Klima and Foissner 2011). Figure 9b-e shows part of a freshly injured cell that was stained 2 d prior to imaging. Interestingly, long-lived compartments bearing AH488HA or FM4-64 still contributed in wound repair. The dynamics of the stained particles near the injured site in Video S4 strongly suggests that the fluorescence of the wound area is not due dye leakage from the external space but to the deposition of labeled organelles.

## Discussion

### Fluid-phase markers are taken up by clathrin-mediated endocytosis in *Chara* branchlet internodal cells

We have previously studied constitutive endocytosis in *Chara* internodal cells with fluorescent styryl dyes like FM1-43 and FM4-64, which are widely used plasma membrane markers (Klima and Foissner 2008). In the present study, we investigated constitutive endocytic pathways in more detail. We were interested in the spatio-temporal dynamics of endocytosis, with a special focus on the uptake of external fluid, which is commonly assumed to occur with all types of endocytic processes. Fluid phase uptake was extensively studied in heterotrophic cells, and it was reported to follow a clathrin-independent endocytic pathway (Etxeberria et al. 2009, 2012). We wondered if in *Chara*, an autotrophic alga, a similar mechanism takes place. For this purpose, we inquired the usability of fluorescent hydrazides as membrane-impermeable, fluid-phase markers in *Chara* internodal branchlet cells, and found that all tested markers were taken up, and could be detected in mobile cytoplasmic organelles. The co-localization with the membrane dye FM4-64 provided proof for the endocytic nature of the internalization process. The uptake was arrested by cold treatment, by ikarugamycin, an inhibitor of clathrin dependent endocytosis, and by methyl-ß-cyclodextrin, which interferes with the sterol composition of the plasma membrane. We have previously shown that inhibition by methyl-ß-cyclodextrin is not due to inhibition of a clathrin-independent pathway but rather reflects the dependence of clathrin-based endocytosis on a specific set of sterols (Hoepflinger et al. 2017). In the present study, we found that fluid-phase markers were internalized in the presence of cytochalasin D, which interferes with actin organization and acto-myosin-dependent motility. These findings are in line with an earlier study showing that the internalization of membrane markers is largely independent of an intact actin cytoskeleton (Klima and Foissner 2008) and consistent with a recent study showing that actin is dispensable for clathrin-mediated events at the plasma membrane of *Arabidopsis* root epidermal cells (Narasimhan et al. 2020).

To sum up, our data suggest that both fluid-phase and plasma membrane are predominantly internalized by a clathrin-dependent and actin-independent mechanism (see also Hoepflinger et al. 2017), even though the existence of a clathrin-independent pathway cannot be fully excluded. The time-dependent distribution and accumulation of fluorescent fluid-phase marker in compartments of the endocytic pathway (TGN, MVBs/PVCs) was confirmed by immunofluorescence and by treatment of cells with wortmannin and BFA. We therefore provide here convincing evidence that FPE is constitutively operating in cells of the autotrophic giant alga *Chara australis* and that fluorescent Alexa hydrazides markers in mM concentrations are well suited to trace extracellular fluid within compartments of the endocytic pathway. Contrary to other studies, however, it was not possible to detect these markers in the cytoplasmic vacuoles or in the central vacuole of *Chara* internodal cells. We hypothesize that the concentration of the fluorescent dyes in these huge compartments was beneath the detection limit.

### AF488HA distributes sequentially to early and late endosomes of the endocytic pathway and reveals very long-lived compartments

In all eukaryotic cells, the endocytic vesicles formed by plasma membrane invagination and subsequent scission build a well-orchestrated dynamic network, that undergoes spatio-temporal progression and maturation (Rink et al. 2005; Scheuring et al. 2011). In order to perform tasks of sorting, processing and recycling, these endocytic structures fuse with each other and/or with pre-existing endosomal compartments. During these events, the luminal cargo designed for degradation or recycling is retained and gradually accumulates within individual endosomes on progressing from early to late status (Rink et al. 2005).

In the present study, initial endocytic events have escaped our observation, since it was not possible to use very short chasing times required for detecting primary endocytic vesicles. On the one hand, incubation times of at least 10 min were necessary, probably because the diffusion of the negatively charged fluid-phase markers was slowed down by the repellent, negatively charged polyanionic pectins, and also size-restricted by the narrow and tortuous pores in the cell wall. The idea that the cell wall acted as a filter barrier is supported by the observation that higher molecular weight AF568HA needed longer incubation times to produce a staining intensity similar to lower molecular weight AF488HA. On the other hand, extensive washing of the cells in dye-free medium was necessary, in order to reduce the residual staining of the cell wall. During this time period the fluorescent markers were distributed to a wide variety of compartments of endocytic origin (see Ueda et al. 2001; Bolte et al. 2004; Klima and Foissner 2008; Hoepflinger et al. 2015 and references therein).

Considering the unusually large size of Chara internodes (here up to 2 cm) and the fact that cytoplasmic organelles, including endosomes are thus conveyed around long distances with the highest known velocity (100 μm.s^-1^), one may ask if the dynamics of cargo transfer between endocytic compartments also shows unusual features. By using concomitant and sequential pulsing with fluid-phase-, membrane- and pH-markers, as well as chasing at varying time intervals, we were able to stain different sub-populations of endosomes, and to reveal interesting characteristics of compartment mixing and maturation. These results, as well as our immunofluorescence data, indicate that AF488HA distributed within 10 min to early (TGN) and after 30 min to late endocytic compartments (MVBs). Notably, these time durations reflect the temporal dynamics of the accumulation of the internalized fluid-phase in early endosomes and of sorting to late compartments, respectively, along the endocytic pathway. This is in agreement with the idea that the TGN temporarily accumulates internalized solutes (Bandmann and Homann 2012; Etxeberria et al. 2012), and that after passing the TGN, endocytic cargo reaches late endocytic compartments (Tanchak and Fowke 1987; Dettmer et al. 2006; Onelli et al. 2008; Bottanelli et al. 2011). Further confirmation came from inhibitor experiments, which showed that fluid-phase markers accumulated in compartments induced by BFA, which consist of Golgi and TGN vesicle aggregates (e.g. Hause et al. 2006; Tse et al. 2006; Naramoto et al. 2014), as well as in wortmannin-induced compartments, which mainly form by homotypic fusion of MVBs (e. g. Jaillais et al. 2006; Tse et al. 2006; Wang et al. 2009; Takac et al. 2012; Foissner et al. 2016).

Interestingly, fluid cargo that was internalized consecutively at time intervals of more than 60 min did not accumulate within the same vesicular structures, nor could it be detected in the vacuole, but it was sorted into spatially distinct late compartments. In animal cells, cargo transfer from early to late endosomes follow a first-order kinetics, with a half time of 10-15 min (Salzman and Maxfield 1988, 1989; Rink et al. 2005). Toyooka et al. (2009) reported that in BY2 cells after 10 to 15 min incubation, the styryl dye FM4-64 co-localized with YFP-SCAMP positive compartments identified as TGN. Furthermore, in *Arabidopsis* leaves, the flagellin sensing membrane receptor FLS2 reached late endosomal compartments after 30 to 50 min of flagellin elicitation (Beck et al. 2012), and in root tips Ravikumar et al. (2018) found that the TGN was stained after about 8-10 min after FM4-64 application. All these time-scales are well comparable to the time scale of endosome-progression found by us in *Chara* in spite of huge cell size differences.

In the course of this study, we revealed the presence of stable, long-lived compartments, which retained their luminal content up to two days after pulse-staining. This finding amends the prevailing view that all endocytic compartments are transitory structures with a high turnover rate. Kang (2014) hypothesized that the plant TGN is likely to be a single-use compartment, which is replenished regularly by membrane trafficking, and Scheuring et al. (2011) suggested that the MVBs/PVCs are non-persistent compartments, that are continuously formed and ultimately consumed through fusion with the vacuole. However, it was also hypothesized that some TGN units may be relatively long-lived (Gendre et al. 2015). Our data are consistent with reports by Etxeberria et al. (2006), who found that fluorescent quantum dots internalized by fluid-phase endocytosis persisted in the cytoplasm of sycamore cultured cells for more than 18 h.

### Late endosomes are involved in plasma membrane repair

In their native habitat, *Chara* internodes often experience injuries due to water currents, animals and pathogen attacks, resulting in wounding of the cell wall and local plasma membrane disruption. In order to maintain their physical and functional integrity, the cells are able to promptly repair the damage, yet the immediate source of membrane and cell wall material for wound healing are poorly characterized. Our group previously reported that at least some of the organelles deposited at the injured site in early stages of wound healing are of putative endosomal nature. In the present study we further substantiate the notion that vesicles of endocytic origin are implicated the membrane restoration process, delivering the material for membrane resealing.

For this study we used local irradiation with strong laser light to damage the plasma membrane and to induce wound healing. Local laser injury of AH488HA-labeled characean internodal cells revealed a strong involvement of organelles containing the fluorescent fluid-phase marker in the process of membrane repair. Cells that were stained for 5 h, and then suspended in dye-free medium reacted with extensive deposition of fluid-phase marker at the wound site, delivered by numerous stably stained compartments from a pool formed well before membrane injury. These results prove an active participation of endocytic organelles in the membrane repair process in *Chara*, in accordance to previous findings (Klima and Foissner 2011). We assume that putative recycling endosomes and probably late endocytic compartments (MVBs) stained with AF488HA were among the repair compartments. Callose was previously found to accumulate at the wound site in *Chara* (Foissner and Wasteneys 2012; Klima and Foissner 2011), and Bohlenius et al. (2010) suggest that callose synthase is loaded via MVBs at papillae formed upon powdery-mildew invasion in barley (see also Gu et al. 2017).

## Supplementary Information

Fig. S1 **Co-localization analysis of BFA-compartments stained with AF488HA and FM4-64.** *Chara* cells (n = 6) were simultaneously pulse-labeled with 2 mM AF488HA and 10 μM FM4-64 for 10 min before treatment with 200 μM BFA for 30 min. The scatterplot illustrates a high degree of co-localization of the two fluorescent signals within BFA-induced compartments (n = 48). The analysis was performed with the PSC plugin of ImageJ, Pearson’s (r_P_) and Spearman’s (r_S_) correlation coefficients are indicated. (PDF 239 kb)

Fig. S2 **Cytochalasin D does not inhibit FPE.** *Chara* cells were treated for 30 min either with (**A**) 100 μM cytochalasin D (CD) or mock-treated with 1 % DMSO in artificial fresh water (**B**), then pulse-stained for 10 min with 2 mM AF488HA in artificial fresh water with corresponding additions of cytochalasin D or, respectively, DMSO. (PDF 331 kb)

Video S1 **Cytoplasmic streaming in an internodal cell of** ***Chara australis*** **observed through a chloroplast-free “window”.** Note detached chloroplasts within the streaming endoplasm. Time elapsed in ms is indicated in the upper left corner. (AVI 4.74 mb)

Video S2 **Punctate fluorescent organelles in the endoplasm of a** ***Chara*** **internodal cell stained with 2 mM AF488HA for 5h.** Time elapsed in ms is indicated in the upper left corner. (AVI 1.43 mb)

Video S3 **Saltatory movements of fluorescent particles in a** ***Chara*** **internodal cell treated with 100 μM cytochalasin D for 30 min and pulse-stained with 2 mM AF488HA for 10 min.** Time elapsed in ms is indicated in the upper left corner. (AVI 0.98 mb)

Video S4 **Wound repair in a** ***Chara*** **internodal cell incubated in 2 mM AF488HA for 5h.** The cell was injured by local irradiation with the 488 nm line of the Ar laser (for details see legend of Fig. 9). Fluorescent structures loaded with AF488HA accumulate at the wound site, appear to fuse with each other and release their cargo. Time interval between frames is 333 ms. (AVI 1.74 mb)
