# Supplementary material for: Fluid-phase and membrane markers reveal spatio-temporal dynamics of membrane traffic and repair in the green alga Chara australis
Source: Protoplasma. 2021 Mar 11;258(4):711–28. doi: 10.1007/s00709-021-01627-z (PMC8211606; doi:10.1007/s00709-021-01627-z)
Supplement: Supplementary file 1 — Co-localization analysis of BFA-compartments stained with AF488HA and FM4-64. Chara cells (n = 6) were simultaneously pulse-labeled with 2 mM AF488HA and 10 μM FM4-64 for 10 min before treatment with 200 μM BFA for 30 min. The scatterplot illustrates a high degree of co-localization of the two fluorescent signals within BFA-induced compartments (n = 48). The analysis was performed with the PSC plugin of ImageJ, Pearson’s (rP) and Spearman’s (rS) correlation coefficients are indicated. (PDF 239 kb) [file 709_2021_1627_MOESM1_ESM.pdf]

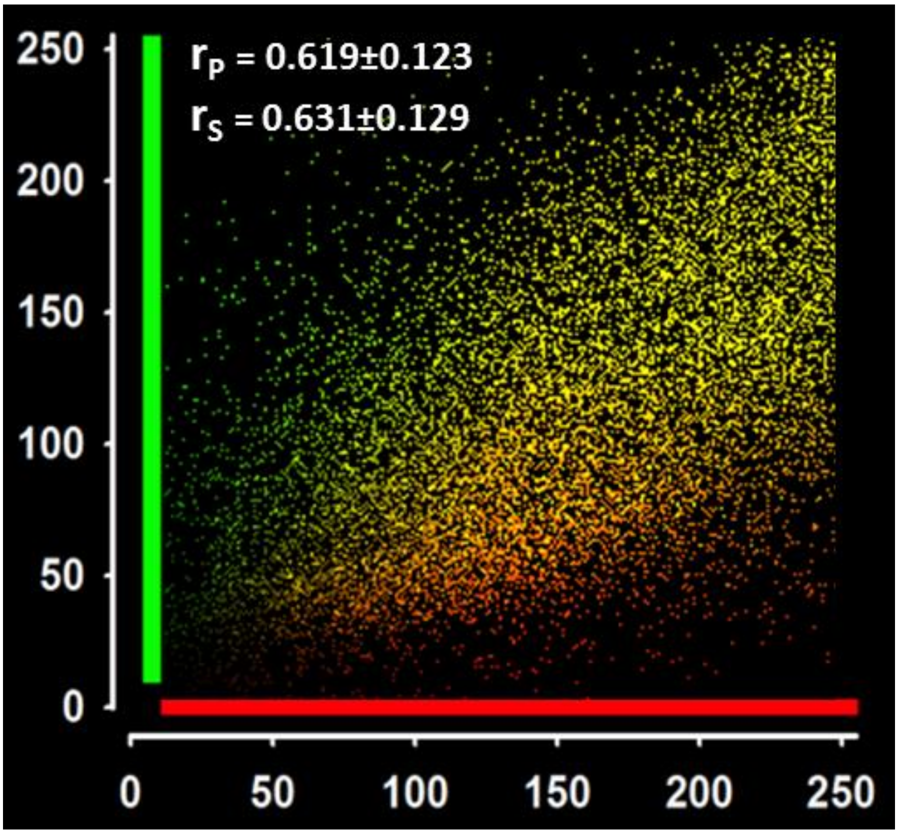

Fig. S1 Colocalization analysis of BFA-compartments stained with AF488HA and FM4-64. *Chara* cells (n = 6) were simultaneously pulse labeled with 2 mM AF488HA and 10 µM FM4-64 for 10 min before treatment with 200 µM BFA for 30 min. The scatterplot illustrates a high degree of co-localization of the two fluorescent signals within BFA-induced compartments (n = 48). The analysis was performed with the PSC plugin of ImageJ, Pearson´s (r_P) and Spearman´s (r_S) correlation coefficients are indicated.
