# Supplementary material for: Fluid-phase and membrane markers reveal spatio-temporal dynamics of membrane traffic and repair in the green alga Chara australis
Source: Protoplasma. 2021 Mar 11;258(4):711–28. doi: 10.1007/s00709-021-01627-z (PMC8211606; doi:10.1007/s00709-021-01627-z)
Supplement: Supplementary file 2 — Cytochalasin D does not inhibit FPE. Chara cells were treated for 30 min either with (A) 100 μM cytochalasin D (CD) or mock-treated with 1 % DMSO in artificial fresh water (B), then pulse-stained for 10 min with 2 mM AF488HA in artificial fresh water with corresponding additions of cytochalasin D or, respectively, DMSO. (PDF 331 kb) [file 709_2021_1627_MOESM2_ESM.pdf]

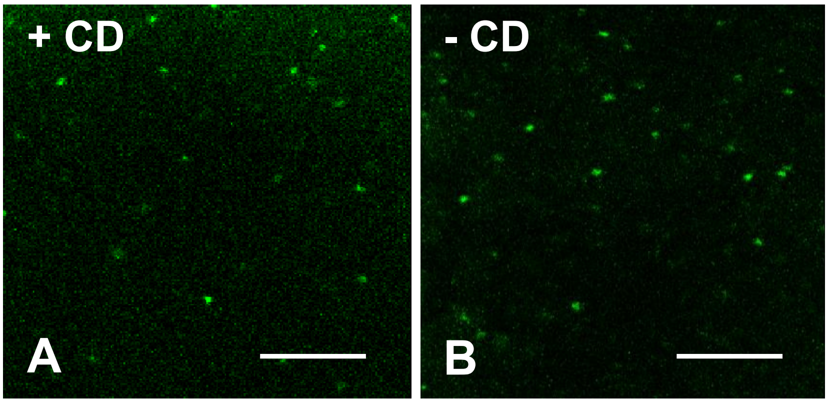

Fig. S2 Cytochalasin D does not inhibit FPE. *Chara* cells were treated for 30 min either with (**A**) 100 µM cytochalasin D (CD) or mock-treated with 1 % DMSO in artificial fresh water (**B**), then pulse-stained for 10 min with 2 mM AF488HA in artificial fresh water with corresponding additions of cytochalasin D or, respectively, DMSO. The images are from time lapse recordings shown in Video S3 and S2, respectively.
